# Comparative analyses of functional traits based on metabolome and economic traits variation of *Bletilla striata*: Contribution of intercropping

**DOI:** 10.3389/fpls.2023.1147076

**Published:** 2023-03-17

**Authors:** Pengfei Deng, Ruoyong Yin, Huiling Wang, Leiru Chen, Xiaoqing Cao, Xiaoniu Xu

**Affiliations:** ^1^ School of Forestry & Landscape Architecture, Anhui Agricultural University, Hefei, Anhui, China; ^2^ School of Architecture & Planning, Anhui Jianzhu University, Hefei, Anhui, China

**Keywords:** intercropping, *Bletilla striata*, functional traits, economic traits, environmental factors

## Abstract

The intercropping practice has been regarded as a practical land-use selection to improve the management benefits of *Bletilla striata* plantations. The reports about the variety of economic and functional traits of Bletilla pseudobulb under intercropping systems were limited. The present study investigated the variation of economic and functional traits of Bletilla pseudobulb under different intercropping systems (the deep-rooted intercropping system: *B. striata* - *Cyclocarya paliurus*, CB; and the shallow-rooted intercropping system: *B. striata* - *Phyllostachys edulis*, PB). The functional traits were analyzed through non-targeted metabolomics based on GC-MS. The results indicated that the PB intercropping system significantly decreased the yield of Bletilla pseudobulb while significantly increasing the total phenol and flavonoids compared with the control (CK). However, there were no significant differences in all economic traits between CB and CK. The functional traits among CB, PB, and CK were separated and exhibited significant differences. Under different intercropping systems, *B. striata* may adopt different functional strategies in response to interspecific competition. The functional node metabolites (D-galactose, cellobiose, raffinose, D-fructose, maltose, and D-ribose) were up-regulated in CB, while the functional node metabolites (L-valine, L-leucine, L-isoleucine, methionine, L-lysine, serine, D-glucose, cellobiose, trehalose, maltose, D-ribose, palatinose, raffinose, xylobiose, L-rhamnose, melezitose, and maltotriose) were up-regulated in PB. The correlation between economic and functional traits depends on the degree of environmental stress. Artificial neural network models (ANNs) accurately predicted the variation in economic traits *via* the combination of functional node metabolites in PB. The correlation analysis of environmental factors indicated that Ns (including TN, NH_4_^+^-, and NO_3_^-^-), SRI (solar radiation intensity), and SOC were the main factors that affected the economic traits (yield, total phenol, and total flavonoids). TN, SRI, and SOC were the main factors affecting the functional traits of the Bletilla pseudobulb. These findings strengthen our understanding of the variation of economic and functional traits of Bletilla pseudobulb under intercropping and clarify the main limiting environmental factors under *B. striata* intercropping systems.

## 1 Introduction

*Bletilla striata* (Thunb.) Rchb. f. (Orchidaceae) is a perennial flowering pseudobulb herb primarily distributed in eastern Asia, including the Yangtze River basin and the Qingling Mountains of China, Japan, Korea, and northern Myanmar (He et al., 2017; Ji et al., 2020). *B. striata* is a shade-tolerant plant that prefers moist and fertile sandy loam soils and is naturally distributed on hillsides, gullies, and sparse woodlands, demonstrating intolerance for photooxidation (Zhang et al., 2019; Jiang et al., 2021). The dried pseudobulb of *B. striata* has been widely applicated in traditional Chinese medicine (TCM) for thousands of years and is officially included in the Pharmacopeia of P. R. China, serving as a hemostatic, detumescent, and body-strengthening agent (Chen et al., 2018a; Ji et al., 2020). It has been documented that the Bletilla pseudobulb contains multiple phytochemical constituents, including polysaccharides, glycosides, bibenzyl, dihydrophenanthrene, phenanthrene, and quinone derivatives (Xu et al., 2019). Modern pharmacological research has revealed that *B. striata* possesses various physiological functions, including hemostatic and wound healing properties, anti-oxidative stress, anti-cancer, anti-viral, and antibacterial properties (He et al., 2017; Ji et al., 2020; Jiang et al., 2021). Due to the valuable applications of *B. striata*, its market demand in medical, pharmaceutical, and cosmetology fields gradually expanded, while its wild resources have been nearly depleted. Artificial cultivation programs are essential for the sustainable development and utilization of *B. striata* resources. Based on its growth habits and requirements of the ecological environment, companion trees or forest resources were attempted intercropping with *B. striata*, which has developed as a critical option and development direction for *B. striata* cultivation in the mountainous areas of eastern China (Zhang et al., 2019).

In recent decades, many researchers have published qualitative and systematic reviews on intercropping. Compared to traditional monoculture, intercropping has shown many advantages, for instance, optimizing crop utilization of natural resources such as solar radiation, water, and nutrients (Brooker et al., 2015; Jensen et al., 2020). Besides, intercropping could help maintain ecological stability by improving pest management, decreasing farmer reliance on pesticides, promoting soil erosion control, and increasing biodiversity (Etchevers et al., 2006; Lopes et al., 2016; Chi et al., 2021). The most crucial advantage of intercropping was that crops could acquire and convert available resources more efficiently, increasing productivity and yield (Gitari et al., 2018; Stomph et al., 2020; Feng et al., 2021). The most common and classic application of intercropping was tall C_4_ cereals intercropped with short C_3_ legumes. The spatio-temporal and functional complementarity of C_3_ and C_4_ plants could improve light and water conversion efficiency (Ton, 2021; Zhang et al., 2022). An additional advantage is the extra nitrogen acquisition from air and soil (Rodriguez et al., 2020).

However, not all intercropping systems demonstrated net favorable (facilitative) interactions (Brooker et al., 2015). Ecologically, the interspecific connections between the coexisting species in the intercropping systems could be described as the antagonistic interactions of competition or positive interactions of mutualism and complementarity (Stomph et al., 2020). Resource complementation implies a reduction in overlap and competition for ecological niches across species, allowing species to access a more excellent range and number of resources in intercropping than in monoculture. Conversely, the overlapping ecological niches may result in the extinction of one species or the separation of ecological niches, ultimately resulting in antagonistic interaction effects (Terradas et al., 2009; Paknia and Schierwater, 2015). On the other side, when plants are stressed, they may accumulate secondary metabolites called “phytoalexins” to withstand external pressures and provide a source of active ingredients for medicinal plants. As a result, medicinal plants may have better quality under moderate environmental stress (Mahajan et al., 2020). Previous studies have shown that medicinal herbs were often subjected to the combined effects of multiple environmental stresses simultaneously during growth, which played a crucial role in accumulating secondary metabolites (Jiang et al., 2020). Excessive stress affects plant resource acquisition, inevitably impacting the plant’s primary metabolism and ultimately altering secondary metabolic accumulation (Guo et al., 2020). Therefore, it is necessary to consider both the yield and the content of medicinal components of *B. striata* under intercropping. Here we used the dry biomass of pseudobulbs to represent the yield of *B. striata* and the total polysaccharides, flavonoids, and phenols to represent the content of medicinal components. We hypothesized that the intercropping groups have better economic traits, including higher yield or medicinal components. The specific results depend on whether intercropping enhance resource sharing and ecological niche complementarity.

Plant functional traits have been extensively employed to characterize plant life history trade-offs, community composition, species interactions, and ecosystem processes (Chelli et al., 2019; Joswig et al., 2022; Walker et al., 2022), especially the trade-off strategy among species in intercropping systems (Zhu et al., 2015; Ajal et al., 2022). The classical and most commonly used plant functional traits could be categorized as soft and hard. Soft traits usually refer to plant traits that are easy to measure quickly, e.g., propagule size, shape, leaf area, SLA, and tree height, while the hard traits refer to traits that more accurately represent the response of plants to external environmental changes, e.g., photosynthetic rate, leaf carbon (C), nitrogen (N), and phosphorus (P) contents (Liu and Ma, 2015). These functional traits could indicate, individually or in combination, the response of an ecosystem to environmental change and strongly affect ecosystem processes (Perez-Harguindeguy et al., 2013; Liu and Ma, 2015). Nevertheless, plant functional traits were not perfect proxies for physiology as a collection of multiple physiological processes in plants, each of which may be differentially limited by evolutionary history and environmental factors. Moreover, the frequency of measurements of classical functional traits was often insufficient to capture variation in plant function on short-time scales. Although plant functional traits were the core of describing plant functions, a large part of unexplained variations in plant ecological processes remains, which limits the possibility of complete insights into plant ecosystem processes.

Plants are exceptional organic chemists, with tens of thousands to hundreds of thousands of tiny compounds in their metabolomes (Kosmacz et al., 2020). These metabolites, the substrate products of enzyme reactions, function as a mechanical link between plant physiology and ecological processes (Ghatak et al., 2018; Carrera et al., 2021; Walker et al., 2022). Briefly, environmental variation over the lifetime of an individual plant will result in modifications to the metabolites, directly affecting its function and adaptation. Consequently, the metabolome is an enormous repository of functional traits of plants. Introducing metabolomes into functional traits may improve the power of functional traits to explain the mechanisms behind plant and ecosystem functioning (Walker et al., 2022). Many reports have convincingly shown how plants respond to abiotic stresses (e.g., temperature, water, light, and nutrient limitation). In general, abiotic stresses rapidly stimulate the production of various signaling molecules, amino acids, proteins, and secondary metabolites (Obata and Fernie, 2012; Ghatak et al., 2018; Singh et al., 2020). However, metabolomics remains at the margins of ecology, and few attempts have been made to integrate metabolomics with plant functional traits, especially in interspecific relationship trade-offs in intercropping systems. Here, we introduced non-targeted metabolomics to plant functional traits to assess differences in functional traits of *B. striata* under different intercropping systems. Due to the differences in the superstructure of intercropping systems, we hypothesized that *B. striata* might adopt different functional strategies to cope with interspecific competition under intercropping systems. We still hypothesized that functional traits could strongly influence the variation of economic traits.

This study established a field experiment with two representative intercropping systems: *B. striata* were intercropped with shallow-rooted bamboo and deep-rooted broadleaf trees, respectively. The operational objectives were to reveal the variations in the response of the economic traits and functional traits of the Bletilla pseudobulb under intercropping. We hope to provide the ecosystem processes information of *B. striata* under intercropping through metabolism-based functional traits. Further analysis of the correlations between environmental factors and economic traits and functional traits of *B. striata* was conducted to identify the limiting environmental factors under intercropping, thus providing reasonable suggestions for the biomimetic cultivation of *B. striata* under forests.

## 2 Materials and methods

### 2.1 Growth conditions, experimental design, and sampling

The field trial of the experiment was located at Qiucun of Guangde City, Anhui, China (31°01′49″N, 119°23′37″E). The fundamental meteorological parameters for the experimental site were compiled from prior investigations. Briefly, the area was classified as a monsoon climate zone in the northern subtropics, with an average annual temperature of 15.4 °C and average annual precipitation of 1328 mm (Chen et al., 2017). The soil type at the site is Alfisol (FAO/UNESCO classification), with a sandy loam texture (Chen et al., 2018b). According to our investigation, the *B. striata* plantation was established in 2012 with 220 hectares, and native subtropical woods and bamboo forests originally covered the experiment sites.

**Figure 1:**
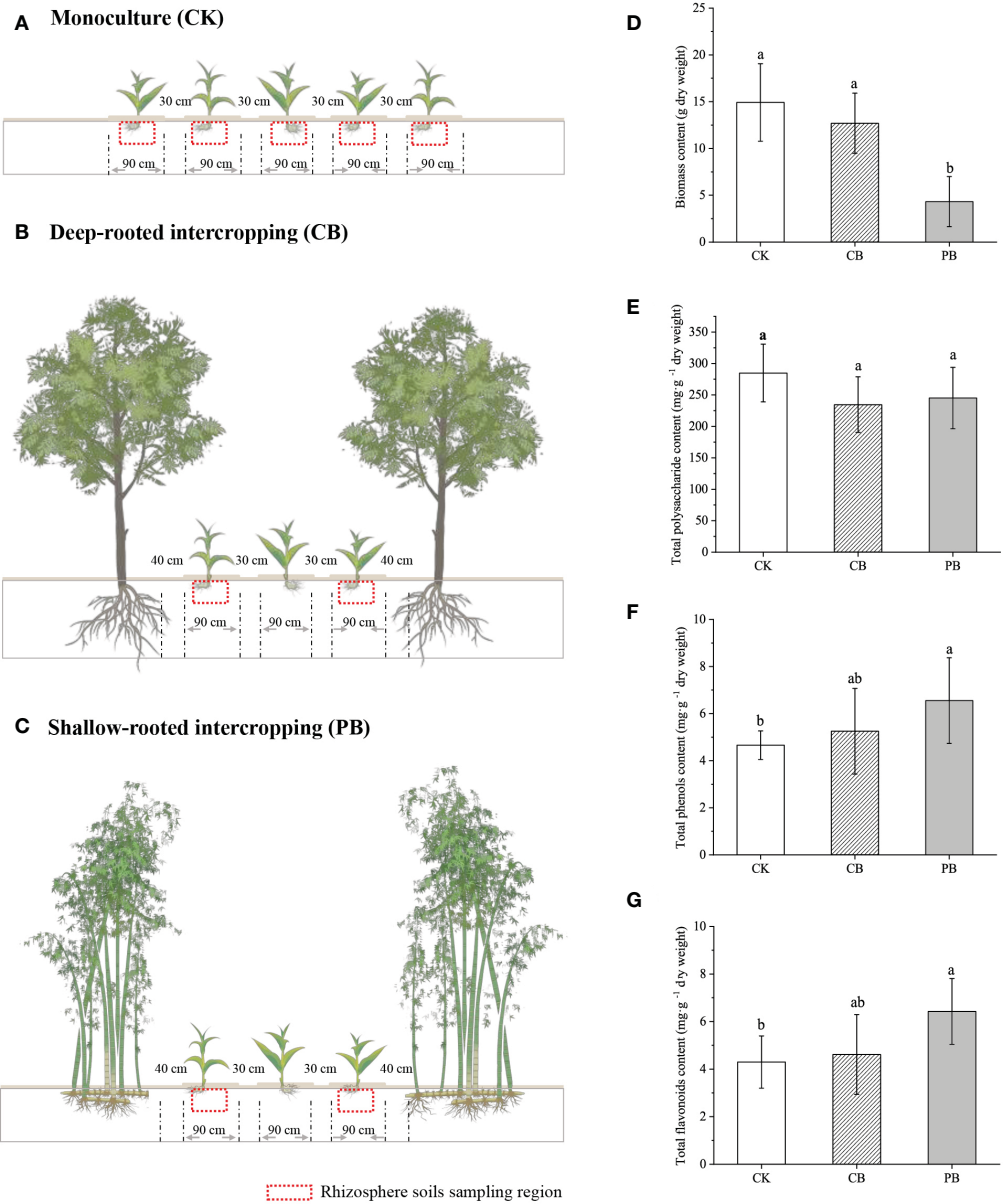
The schematic of *B. striata* cultivation modes: **(A)** monoculture, **(B)** *C. paliurus* intercropping with *B. striata*, **(C)** Moso bamboo intercropping with *B. striata*. The economic traits of *B. striata* variations among monoculture and intercropping modes: **(D)** biomass content, **(E)** total polysaccharide, **(F)** total phenol, and **(G)** total flavonoids, different small letter in the same column indicates significant difference (*p* < 0.05) among groups.

The intercropping experiment field was established in April 2018, containing *Phyllostachys pubescens* (Moso bamboo) intercropped with *B. striata* (PB) and *Cyclocarya paliurus* intercropped with *B. striata* (CB), which represents shallow root and deep root companion species intercropping with *B. striata*, respectively. *C. paliurus* was planted during the establishment of the plantation and has been cultivated for eight years. It currently stands at a height of approximately 4 m. *Ph. pubescens* is the native forest that has been modified through thinning to form a belt, and it currently stands at around 12 m. The adjacent *B. striata* monoculture plantation was considered as control (CK). In the experimental intercropping plantation, the *B. striata* were planted on parallel strips (30 m long, 0.9 m wide, 0.3 m high), and each strip was 0.3 m wide apart, with a plant density of 160,000 ha ^-1^. For every three *B. striata* planting strips, a parallel strip of *Ph. pubescens* or *C. paliurus* was set aside (1 m wide), with a planting density of 3000 and 600 per hectare, respectively (**Figures 1B, C**). The planting pattern of CK was identical to those for intercropping (**Figure 1A**). The *B. striata* in all plantations employed the same management (e.g., planting age, density, irrigation, and pest control), and the *B. striata* had been planted for three years and had yet to be harvested before the sampling.

The samples were collected on October 20, 2020. For each plantation, *B. striata* were collected from 6 adjacent strips on an S-shaped pattern, resulting in 6 replicates per treatment (each replicate was formed by merging three individuals of *B. striata*). There were 18 replicates in total (6 replicates/PB + 6 replicates/CB + 6 replicates/CK). The environmental variables, including rhizosphere soils and solar radiation intensity (SRI) of *B. striata*, were sampled and recorded paired with each plant sample. The plant samples were fresh with dry ice, delivered to the laboratory immediately, and stored at -80°C for further non-targeted metabolomics and phytochemical measurements. The soil samples were air-dried to determine soil physicochemical properties, concluding soil pH, soil water content (SWC), soil organic carbon (SOC), total nitrogen (TN), total phosphorus (TP), and soil available nutrients, including ammonium-nitrogen (NH_4_^+^-N), nitrate-nitrogen (NO_3_^-^-N), available potassium (AK), and available calcium (ACa).

### 2.2 Environment variables analysis

The contents of SOC and TN were determined by CN Analyzer (EA 3000, Vector, Italy) using 10 mg of dried powder samples. Soil samples for TP were pretreated by Micro-Kjeldahl digestion (HNO_3_: HClO_4_ at 3:1) and determined using a flow injection auto-analyzer (FIA Star 5000, FOSS, Denmark). The NH_4_^+^-N and NO_3_^-^-N were extracted by 50 mL of 1 mol L^-1^ KCl from 30-g fresh soil samples and determined by a flow injection auto-analyzer (FIA Star 5000, FOSS, Denmark). The AK, ACa, and AMg were extracted by 100 mL of 1 mol L^-1^ NH_4_Cl from 0.5 g dried soil samples and determined by an atomic absorption spectrophotometer (AA-6300, Shimadzu, Japan). The solar radiation intensity (SRI) was measured with an illuminance meter (TES-1339, TES, China), and the value was recorded at 2:00 p.m., 30 cm above the top of the *B. striata*. In this study, soil chemical properties and SRI were regarded as environmental information, and the results were presented in **Table 1**.

**Table 1:** Environmental factors properties among *B. striata* plantations.

Treatment	SRI	SWC	pH	SOC	TN	TP	AK	ACa	NO_3_^-^-N	NH_4_^+^-N
	Lux	%		(g Kg ^-1^)	(g Kg ^-1^)	(g Kg ^-1^)	(mg Kg ^-1^)	(mg Kg ^-1^)	(mg Kg ^-1^)	(mg Kg ^-1^)
CK	88110±958a	15.49±2.77b	4.96±0.38a	10.28±0.50a	0.63±0.04a	0.39±0.05a	0.25±0.02a	0.38±0.06a	0.12±0.01b	2.06±0.32b
CB	62394±558b	19.37±0.78a	4.73±0.28a	9.04±0.91a	0.56±0.05a	0.35±0.11a	0.27±0.03a	0.27±0.17a	0.26±0.08a	1.34±0.20c
PB	56607±543c	17.15±1.13ab	4.89±0.17a	6.45±1.13b	0.44±0.06b	0.44±0.20a	0.26±0.02a	0.39±0.19a	0.09±0.02b	2.48±0.29a

CB: the intercropping plantation (Cyclocarya paliurus with B. striata), PB: the intercropping plantation (Moso bamboo with B. striata), CK: the control (monoculture plantation). SRI, solar radiation intensity; SWC, soil water content; SOC, soil organic carbon; TN, total nitrogen; TP, total phosphorus; AK, available potassium; ACa, available calcium; NH_4_^+^-N, ammonium-nitrogen; NO_3_^-^-N, nitrate-nitrogen The different letters in the same column indicate a significant difference among groups by one-way ANOVA (LSD, *p* < 0.05).

### 2.3 Determination of functional traits based on metabolome

#### 2.3.1 Metabolites extraction and derivatization

The 20-mg freeze-dried powder samples were transferred into a 2 mL tube and soaked in an extraction solvent containing 450 μL of 80% methanol and 10 μL of 0.5 mg mL ^−1^ internal standard adonitol. Samples were vortexed for 30 s and homogenized with a tissue grinder for 4 min at 35 Hz to adequately extract the metabolites. The samples were centrifuged at 4 °C for 15 min at 10000 rpm, then 260 μL of the supernatant was transferred to a fresh tube. The QC samples were combined from 50 μL of each sample. After evaporating in a vacuum concentrator for 6 h, the 50 μL of 20 mg mL ^−1^ methoxyamination hydrochloride in pyridine was added and then incubated at 80 °C for 30 min. The Silylation reaction was performed by adding 70 μL of derivatization reagents (99% BSTFA+1% TMCS; Supelco) at 70 °C for 1.5 h. After gradually cooling the samples to room temperature, 3 μL of 500 mg mL ^−1^ alkane in hexane (C7-C40; Anpel) was added to the QC samples. Finally, all samples were analyzed by gas chromatography coupled with a mass spectrometer (GC-MS). The raw data of metabolites were deposited in the MetaboLights database (Accession Number: MTBLS4752).

#### 2.3.2 Metabolite profiling based on GC-MS

The metabolite profiling was performed using a Thermo triple quadrupole tandem MS (TRACE1310 & TSQ8000, Thermo Scientific, United States). The GC separations were carried out using a TG-5SILMS (Thermo Scientific, United States) capillary column (30 m× 0.25 mm× 0.25 µm). Sample volumes of 1 µL were injected with a splitless mode. The front injection temperature was set at 280 °C, the transfer line temperature was set to 280 °C, and the ion source temperature was set at 260 °C, respectively. Helium was the carrier gas with a 1 mL min ^−1^ constant flow rate. The GC temperature program was set isothermally at 50 °C for 1 min, ramped from 50 to 310 °C at a rate of 5 °C min ^−1^, and finally held at 310 °C for 6 min. All mass spectra were acquired in electron impact ionization (EI) mode (70 eV) with a full-scan range of 50-550 m/z at 9 scans per second after a solvent delay of 9 min.

### 2.4 Determination of economic traits

#### 2.4.1 Determination of total polysaccharide content

The total polysaccharide content was determined using the Phenol-Sulfuric acid method, and the absorbance was measured at 490 nm with a UV-visible spectrophotometer (Lambda 750s, PerkinElmer, United States). The standard curve was prepared using glucose as the x-axis and absorbance as the y-axis. The linear regression equation obtained was: , with an R² value of 0.999.

*B. striata* polysaccharides were extracted using the ethanol precipitation method, following the procedure of Lu et al. (2021). Powdered samples (100 mg) were dissolved in pure water at a material ratio of 1:50 (*w*/*v*) and refluxed at 90 °C for 2 h. The extraction was repeated twice, and the extracts were combined. The 95% ethanol was added to the extract with the extract ratio of ethanol 1:5 (*v*/*v*) for precipitation. The precipitate was centrifuged at 5000 rpm for 6 min after storage in a refrigerator at 4 °C for 12 h. The resulting precipitate was diluted to 10 mL with pure water, and 1 mL of the diluted solution was used to calculate the total polysaccharide content using the standard curve.

#### 2.4.2 Determination of total flavonoids content

The aluminum nitrate colorimetric method was used to determine the total flavonoids content. The absorbance was measured at 510 nm using a UV-visible spectrophotometer. The standard curve was prepared using Rutin (≥ 95.0%; Aladdin) as a reference, with the linear regression equation obtained as , and an R² value of 0.999, where Rutin was the x-axis and absorbance was the y-axis.

Following a previously reported method with slight modifications (Lu et al., 2021), the samples were precisely weighed at 300 mg and dissolved in 90% methanol with the material ratio to liquid 1:50 (*w*/*v*). The ultrasonic extraction parameters were set at 640 W for 25 min. The supernatant was collected after centrifugation at 4000 r min ^−1^ for 15 min. The extraction was repeated once more, and the extracts were combined. The supernatant was evaporated, and the volume was adjusted to 10 mL with 90% methanol. From the extraction solution, 1 mL was taken, and the total flavonoids content was calculated using the standard curve.

#### 2.4.3 Determination of total phenol content

The total phenol content was determined using the Folin-Ciocalteu method. The standard curve was prepared using gallic acid (≥ 95.0%; Aladdin) as a reference, with the linear regression equation obtained as y = 5.3502x+0.017, and an R² value of 0.999, where gallic acid was the x-axis and absorbance was the y-axis. The extraction method for total phenol was the same as that for total flavonoids, and total phenol content was calculated using the standard curve.

### 2.5 Statistical analysis

The pre-processing of GC-MS raw tags was conducted in Tracefinder software. Data cleaning processes were calculated on the R package “MetaboAnalyst”. The clean data matrix was imported into SIMCA-P software for multivariate statistical analysis. The differential metabolites were screened based on the partial least squares-discriminant analysis (PLS-DA) model according to the thresholds of VIP > 1 and *p* < 0.05. The National Institute of Standards and Technology (NIST) mass spectra library identified most differential metabolites based on mass spectral matching. The *n*-alkanes retention index (RI) of metabolites was further qualitatively identified based on the RI provided by the Golm Metabolome Database (GMD). The KEGG Pathway Analysis module on the MetaboAnalyst platform calculated differential metabolic pathways, and *Arabidopsis thaliana* was selected as a reference.

The permutational multivariate analysis of variance (PERMANOVA) was employed to detect the significant differences among groups in the functional traits. The correlation between environmental variables and economic trait components was conducted using the Spearman correlation coefficient and the R package “pheatmap” for visualization. The Mantel test calculated the correlation between environmental variables and functional traits, and environmental variables were presented in the distance-based redundancy analysis (db-RDA). The variation partitioning analysis (VPA) module of the R package “vegan” was employed to quantify the relative contributions of environmental variables to functional traits. The chemical and biochemical relationships of metabolite-metabolite relationships were calculated from online databases (http://metamapp.fiehnlab.ucdavis.edu/), and the interaction network was constructed using Cytoscape software (Guan et al., 2021). The artificial neural networks (ANNs) were performed using the R package “neuralnet”, which contained a three-layered (input layer, hidden layer, and output layer) BP network topology structure (Liu et al., 2020).

## 3 Results

### 3.1 Variation of economic traits under intercropping

The economic traits of Bletilla pseudobulb (e.g., yield, total polysaccharide, total flavonoids, and total phenol) were investigated (**Figure 1**). The result showed that the pseudobulb yield (dry biomass) significantly (*p* < 0.05) decreased in the PB group than in CK and CB groups. In contrast, the CK and CB groups exhibited no significant difference. The content of total polysaccharides showed no significant differences among the three modes. The PB group had the highest total flavonoids and phenol concentration, averaging 6.55 mg g ^−1^ and 5.07 mg g ^-1^ (DW), respectively. Overall, significant differences (*p* < 0.05) in yield, as well as main medicinal components (total phenol, total flavonoids), were observed between PB and CK. On the contrary, no significant differences existed in economic traits between CB and CK.

### 3.2 Variation of functional traits profiles under intercropping

**Figure 2:**
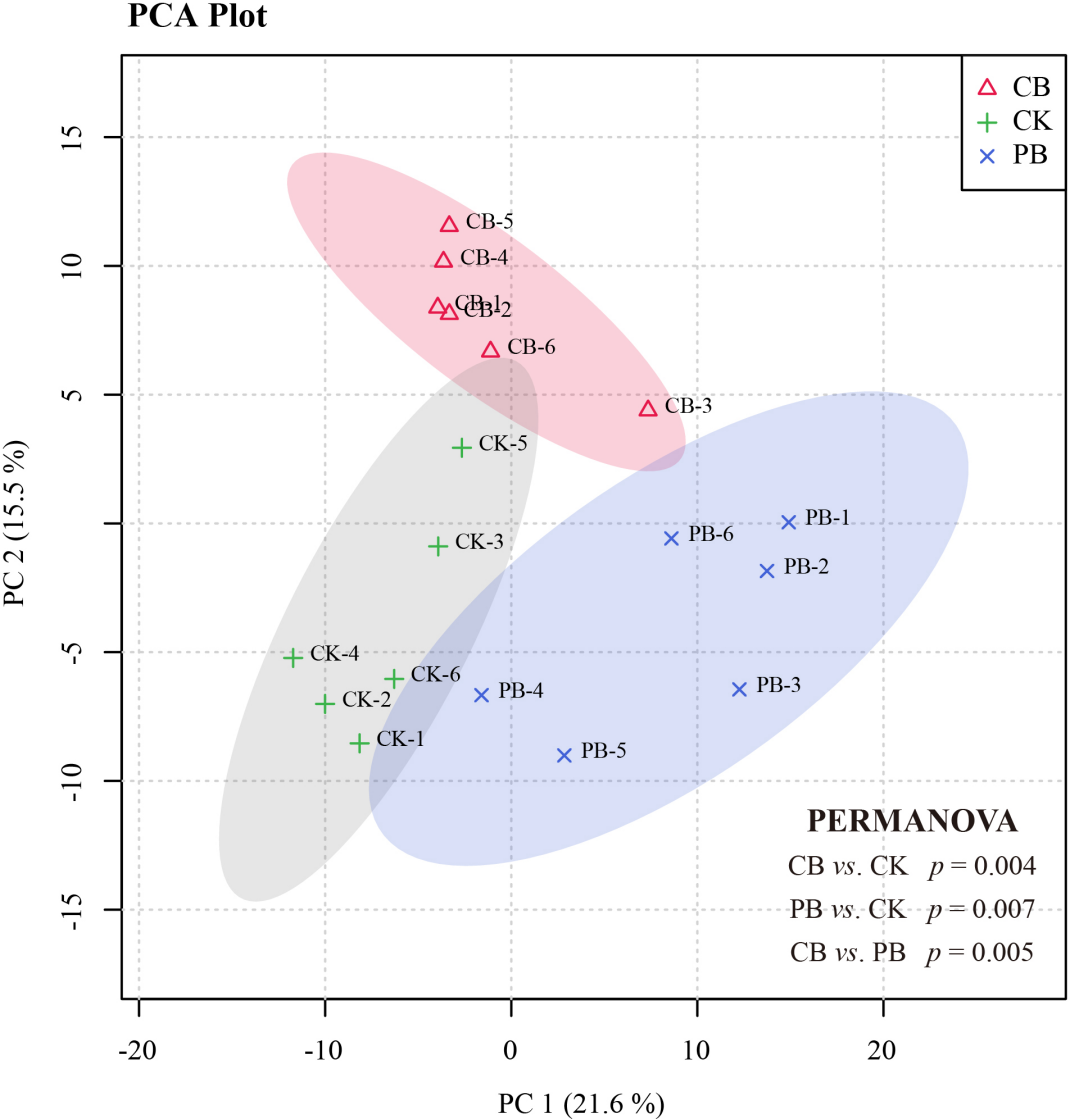
PCA score scatter plot derived from the functional traits for all samples: *C. paliurus* intercropping with *B. striata* (CB), Moso bamboo intercropping with *B. striata* (PB), and monoculture (CK).

The PCA score plots were established to observe the functional traits profile variations among PB, CB, and CK groups. All samples from groups were contained in the 95% confidence interval (**Figure 2**). The PCA separated the functional traits profile of all cultivation modes into three groups. Subsequence PERMAVONA analysis further confirmed the significant differences among the three groups in statistics (*p* < 0.05 for each group; **Figure 2**). The PLS-DA models with UV scaling were established to probe into the differential metabolites of Bletilla pseudobulb under intercropping *vs*. monoculture. Based on the first principal component, the PLS-DA score plots of PB *vs*. CK and CB *vs*. CK showed great separation from CK (**Figures S1A, C**). The parameters of the cross-validation plot permutation (*n* = 200) tests were evaluated to test the accuracy of CB vs. CK mode (*R* ^2^ = 0.992, *Q* ^2^ = 0.222; **Figure S1B**) and PB *vs*. CK mode (*R* ^2^ = 0.969, *Q* ^2^ = 0.199; **Figure S1D**), and the results indicated that the models were credible with no evidence of overfitting.

**Figure 3:**
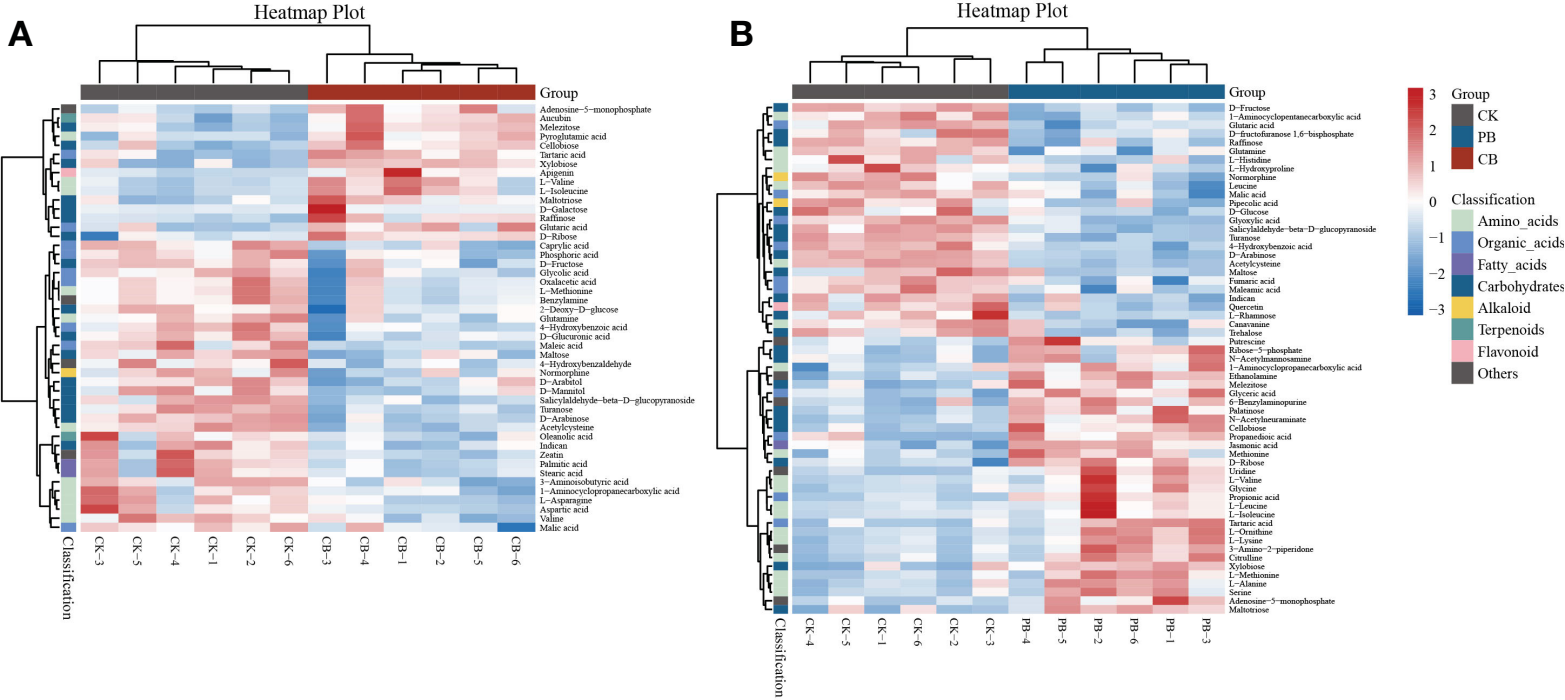
Heat map visualization of identified differential metabolites from the Bletilla pseudobulb in intercropping *vs*. monoculture. **(A)** indicates the *C. paliurus* intercropping with *B. striata* (CB) vs. Control and **(B)** indicates the Moso bamboo intercropping with *B. striata* (PB) vs. Control.

The differential metabolites of CB vs. CK and PB vs. CK were screened based on the PLS-DA model, and we observed that the total 47 differential metabolites (VIP > 1 and *p* < 0.05) were annotated in CB vs. CK, with 20 and 27 being up- and down-regulated, respectively. In the comparison groups of PB vs. CK, a total of 59 differential metabolites (VIP > 1 and *p* < 0.05) were identified, including that 29 and 30 were up- and down-regulated, respectively (**Table S1**). The heatmaps were generated to acquire an outline of differential metabolites among the samples (**Figure 3**). All groups’ differential metabolites were mainly categorized as amino acids and derivatives, organic acids, fatty acids, carbohydrates, flavonoids, and alkaloids. The differential metabolites were much more in PB groups than in CB groups, indicating that the PB intercropping had a more significant effect on the functional traits of the pseudobulb of *B. striata*.

### 3.3 Enrichment analysis of differential metabolites in KEGG metabolic pathways

**Figure 4:**
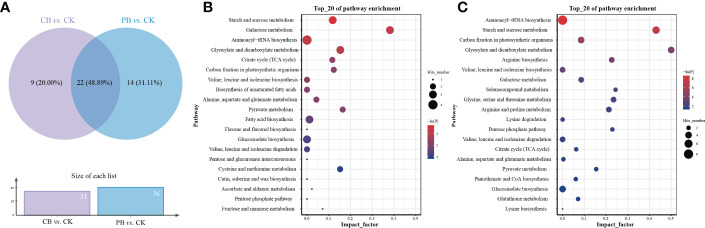
Enrichment result of differential metabolites in KEGG metabolic pathways. **(A)** Venn diagram showing the unique and shared differential metabolites from the Bletilla pseudobulb of intercropping *vs*. monoculture. The bubble map of metabolite pathway enrichment analysis in the Bletilla pseudobulb of intercropping *vs*. monoculture, **(B)** indicates the *C. paliurus* intercropping with *B. striata* (CB) vs. Control; **(C)** indicates the Moso bamboo intercropping with *B. striata* (PB) vs. Control.

The related metabolic pathway analysis was carried out on the differential metabolites of two pairwise comparison groups using the Pathway Analysis module of MetaboAnalyst platform retrieval from KEGG. The pathway enrichment results (**Table S2**) showed that 31 and 36 differential metabolic pathways were enriched in CB vs. CK and PB vs. CK, respectively. The distribution of differential metabolic pathways in the Venn diagrams (**Figure 4A**) demonstrated that CB vs. CK and PB vs. CK groups shared the most differential metabolic pathways, indicating that the two intercropping systems encompass a common impact on the metabolism of Bletilla pseudobulb. Besides, the PB vs. CK group had a more abundant unique differential metabolic pathways relative to CB vs. CK, indicating that PB intercropping exerted a more significant effect on the functional traits of Bletilla pseudobulb.

Among all of these differential metabolic pathways, 3 significant (*p* < 0.05 and -ln(*p*) > 1; **Figure 4B**) differential metabolic pathways were enriched in the comparison group of CB vs. CK, including starch and sucrose metabolism, galactose metabolism, aminoacyl-tRNA biosynthesis, and glyoxylate and dicarboxylate metabolism. In the comparison group of PB vs. CK, 6 significant (*p* < 0.05 and -ln(*p*) > 1; **Figure 4C**) differential metabolic pathways were enriched, including aminoacyl-tRNA biosynthesis, starch and sucrose metabolism, carbon fixation in photosynthetic organisms, glyoxylate and dicarboxylate metabolism, arginine biosynthesis, and valine, leucine, and isoleucine biosynthesis. In addition, 2 significant (*p* < 0.05 and -ln(*p*) > 1) differential metabolic pathways were found in both CB vs. CK and PB vs. CK groups, including aminoacyl-tRNA biosynthesis and starch and sucrose metabolism.

### 3.4 Metabolite-metabolite interaction (MMI) networks analysis

**Figure 5:**
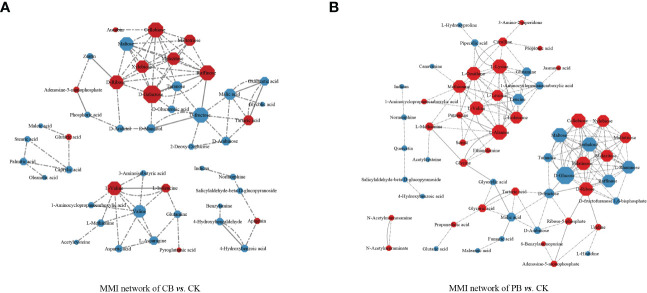
The metabolite-metabolite interaction network (MMI) of significantly different metabolites that participated in KEGG pathways. **(A)** indicates the *C. paliurus* intercropping with *B. striata* (CB) vs. Control and **(B)** indicates the Moso bamboo intercropping with *B. striata* (PB) vs. Control. The node indicates the correlated metabolisms in these networks, and the lines between the two nodes represent the biological relationships between two metabolites. The size of the node indicates the degree value, and the color indicates the log_2_ FC (red indicates up-regulated and blue indicates down-regulated).

To obtain information on the interaction network of intercropping-responsive metabolites involved in KEGG pathways and determine the critical metabolites, the interaction network of differential metabolites was developed in the Cytoscape. The results revealed that the metabolites interacted and coordinated the regulation of multiple metabolic pathways. Almost all metabolites were presented in the same metabolic pathway in these metabolites in CB vs. CK (**Figure 5A**) and PB vs. CK (**Figure 5B**). However, the intercropping-responsive differential metabolites in the groups of PB vs. CK showed a more complex interaction, and comprehensive metabolite network in the MMI network compared to CB vs. CK.

In the MMI network of CB vs. CK, in terms of the complexity of the interactions (degree value), the carbohydrates (D-galactose, cellobiose, raffinose, D-fructose, maltose, and D-ribose) and amino acids (L-valine) played essential roles. In the MMI network of PB vs. CK, the carbohydrates (D-glucose, cellobiose, trehalose, maltose, D-ribose, palatinose, raffinose, xylobiose, L-rhamnose, melezitose, and maltotriose) and amino acids and derivatives (L-alanine, L-lysine, L-ornithine, L-valine, methionine, L-isoleucine and L-leucine, and leucine) exerted significant effects on the change in metabolite levels in terms of the complexity of the interactions. The amino acids and carbohydrates seem to be the functional nodes role of the MMI networks. In the CB vs. CK, carbohydrates were the functional nodes of the MMI network. In contrast, carbohydrates and amino acids were the functional nodes in the PB vs. CK, which indicates that *B. striata* have adopted different metabolic approaches and coping strategies under different intercropping systems.

### 3.5 Factors affecting the economic traits and functional traits under intercropping

**Figure 6:**
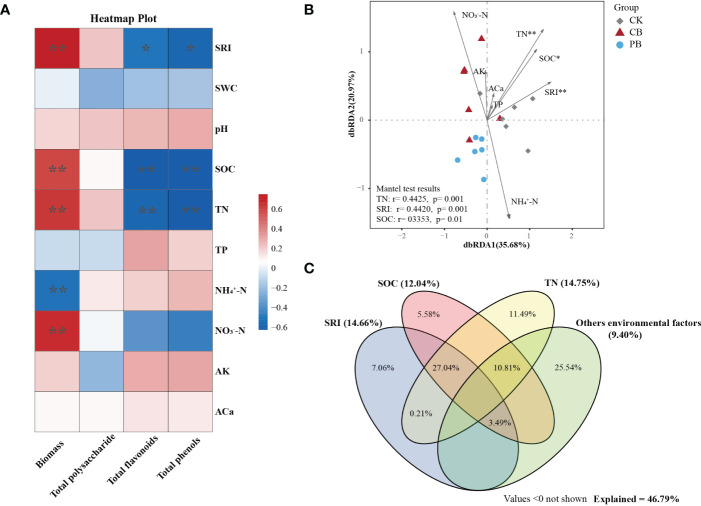
Effects of environmental factors on economic and functional traits. **(A)** The correlation (Spearman) between environmental factors and economic character of Bletilla pseudobulb. **(B)** The metabolic profiles of Bletilla pseudobulb were demonstrated by distance-based redundancy analysis (db-RDA), using environmental factors as explanatory variables; CB: C. paliurus intercropping with B. striata, PB: Moso bamboo intercropping with B.striata, CK: control group. **(C)** Variance partitioning analysis (VPA) of the effects of environmental factors on metabolic profiles of Bletilla pseudobulb. * p <0.05 and ** p <0.01.

The Spearman correlation analysis between environmental factors and economic traits of Bletilla pseudobulb was calculated (**Figure 6A**). The dry biomass has a significant positive correlation with SRI (*r* = 0.74, *p* = 0.001), SOC (*r* = 0.72, *p* = 0.009), TN (*r* = 0.77, *p* = 0.001), and NO_3_^-^-N (*r* = 0.65, *p* = 0.003), while has a significant negative correlation with NH_4_^+^-N (*r* = -0.63, *p* = 0.005). Both the total flavonoids and total phenol showed a significant negative correlation with SRI (*r* = -0.51, *p* = 0.029; *r* = -0.59, *p* = 0.010), SOC (*r* = -0.72, *p* = 0.001; *r* = -0.67, *p* = 0.002), TN (*r* = -0.62, *p* = 0.006; *r* = -0.61, *p* = 0.007), respectively. Interestingly, the total polysaccharide showed no significant correlation with environmental factors. The environmental factors, SRI, SOC, and Ns (TN, NO_3_^-^-N, and NH_4_^+^-N), were the main elements controlling the variation of economic traits of Bletilla pseudobulb under intercropping systems.

The environmental factors were taken as explanatory variables to determine the correlation with functional traits of Bletilla pseudobulb under intercropping. The results of the Mantel test revealed a positive significant relationship between the functional traits and environmental factors, i.e., TN (*r* = 0.44, *p* = 0.001), SRI (*r* = 0.44, *p* = 0.001), and SOC (*r* = 0.34, *p* = 0.01). Additionally, the db-RDA ordination plots showed that the dbRDA1 and dbRDA2 axis explained 35.68% and 20.97% of the total variation in the composition of functional traits, respectively (**Figure 6B**). The db-RDA-based VPA was employed to quantify the contributions of environmental factors on functional traits (**Figure 6C**). According to the VPA result, all the environmental factors explained 46.79% variation of metabolic profiles. TN had a more significant contribution (14.75%) than SRI (14.66%) and SOC (12.04%).

### 3.6 Correlation analysis between the economic traits and functional traits

**Figure 7:**
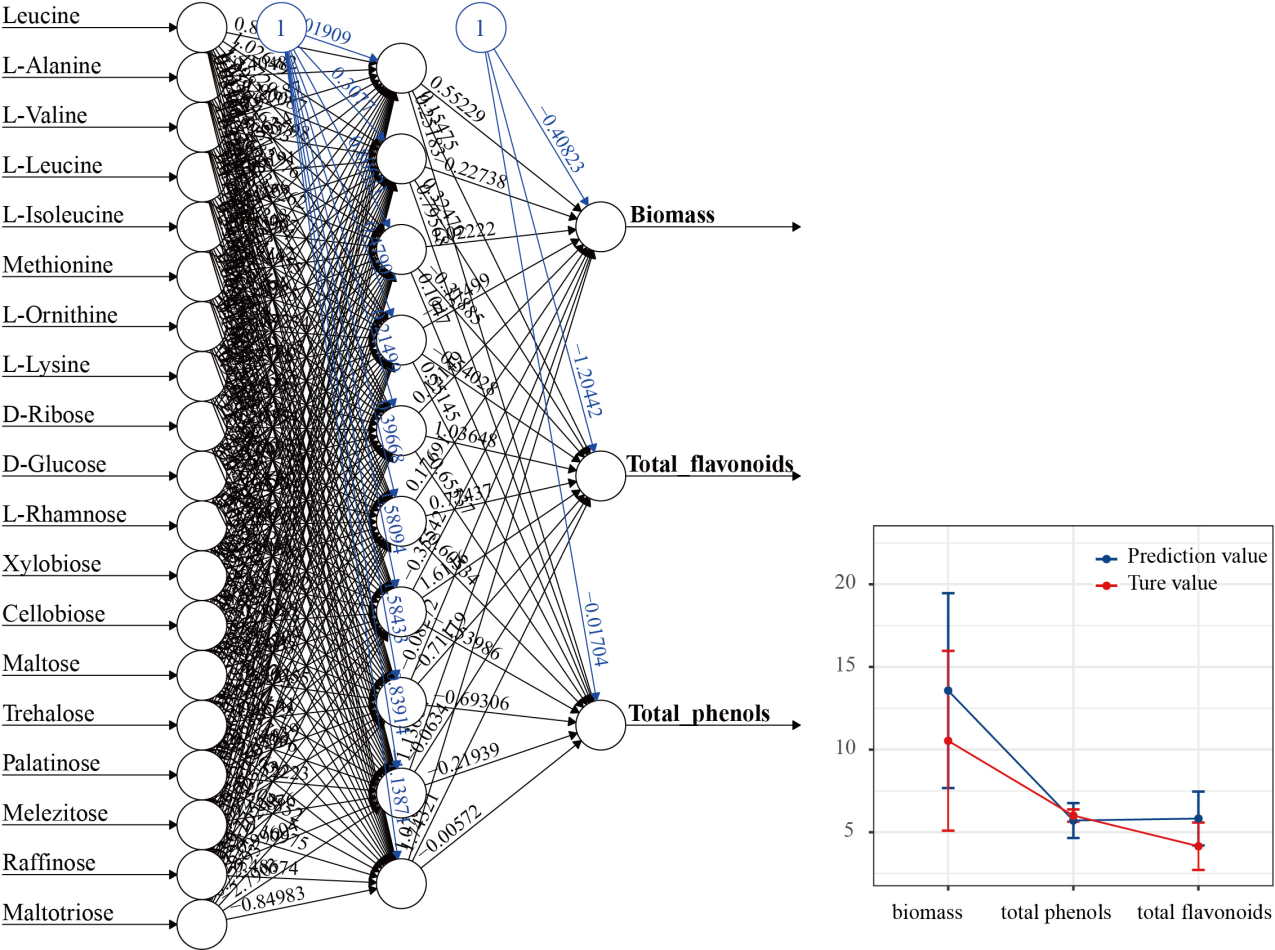
Artificial neural network topology.

Spearman correlation analysis was employed to calculate the correlations between functional node metabolites of MMI networks metabolites and economic traits (**Table S3**). The result indicated that all functional node metabolites had no significant correlation with economic traits in CB. In contrast, a high correlation was observed between functional node metabolites and economic traits in PB. The amino acid metabolites were mainly negatively regulated biomass and total polysaccharide while positively affecting total flavonoids and phenol. Carbohydrates metabolism mainly positively regulates total flavonoids and total phenol. Traditional prediction methods, such as multiple linear regression, cannot produce valid estimates in the presence of multicollinearity and the absence of linear correlation, but the ANN model does not require any link between the input and output variables. The ANN modes (**Figure 7**) were established to estimate whether functional node metabolites could explain the variations of economic traits in PB mode, using functional nodes as input (predictive) variables and differential economic traits (biomass, total flavonoids, and total phenol) as the output variable. The multilayer perceptron (MLP) feedforward network was based on 19 inputs (including 8 amino acids and 11 sugars), 10 hidden layers, and 3 outputs. ANN prediction results indicated that the functional nodes metabolites of PB provided reliable predictions for the biomass, total flavonoids, and phenol variation, and the correlation coefficients are 0.987, 0.998, and 0.951, respectively.

## 4 Discussions

Traditional Chinese medicine (TCM) is a particular crop. Along with the yield of the target crop, the concentrations of secondary metabolites are critical for determining the efficacy and quality of the TCM. As a consequence, the objective of optimal TCM cultivation is to maximize both growth and secondary metabolite contents. Although genetic processes control the generation of active components in TCM, ontogeny, morphogenesis, environmental variables, and agricultural practices also influence secondary metabolite biosynthesis (Yang et al., 2018; Mohammadi Bazargani et al., 2021). Among them, intercropping is an effective agricultural practice that affects the yield and the accumulation of secondary metabolites by changing the interspecific competition or complementarity among crop individuals. In this study, two different intercropping modes were established. The result indicated that intercropping could impact the environmental factors in the plantation through the interspecific interactions of *B. striata* with the companion species (upper forest), consequently affecting the economic and functional traits of Bletilla pseudobulb.

### 4.1 Intercropping effects on the economic traits of Bletilla pseudobulb

Intercropping affects environmental factors such as light and soil physicochemical properties in the habitat through interspecific competition or complementarity. Habitat environments were considered critical factors affecting gene expression in the biosynthetic pathway of secondary metabolites in TCM (Mohammadi Bazargani et al., 2021). Although various abiotic stress resulting from plant individual interactions in intercropping could affect the content and quantity of secondary metabolites in TCM, correlations between secondary metabolite levels and external variables may reflect the degree of variation and adaptation to specific environment (Sampaio et al., 2016; Ngwene et al., 2017; Demasi et al., 2018; Li et al., 2020). Different from our hypothesis, the economic traits of *B. striata* exhibited different responses in different intercropping systems, indicating that *B. striata* faced different degrees of interspecific competition under intercropping systems.

In the present study, the PB intercropping showed a significant decrease in yield (dry biomass) and a significant increase in quality (total flavonoids and total phenol). These could be related to decreased SRI and soil resources (SOC, TN, NO_3_^-^-N, and NH_4_^+^-N) in the PB intercropping system resulting from intense interspecific competition between Moso bamboo and *B. striata*. Moso bamboo was considered a dominant species in the interspecific competition, and it was also recognized as an invasive species in the ecological and environmental field (Lima et al., 2012; Shinohara et al., 2019; Xu et al., 2020). The clonal growth habit was critical in promoting competition and invasion of Moso bamboo (Xu et al., 2020). Moso bamboo rhizome has rapid growth and extension (Makita, 2005), thus structuring a vast bamboo rhizome system, forming a rapid distribution front in the forest, and creating solid competition with the neighboring communities. Moso bamboo has greater fine root biomass and total root length than broad-leaved trees, and its fine root development and turnover rates are more extensive than those of broad-leaved trees (Liu et al., 2013). For these reasons, Moso bamboo could change the soil nutrient status through the rapid growth of bamboo rhizomes and greater biomass (Okutomi et al., 1996). A previous study showed that the expansion of Moso bamboo decreased the soil content of SOC and TN (Bai et al., 2016), which was consistent with our findings. Soil nutrient deficiencies, especially N, limit most ecosystems’ total and net primary productivity, thus decreasing the yield of Bletilla pseudobulb. Song et al. (2013) reported that expansion of the Moso bamboo strengthened ammonification and reduced nitrification, thus having the advantage in the ratio of soil NH_4_^+^-/NO_3_^-^ and the soil NH_4_^+^-N content. However, the increased NH_4_^+^-N content in PB intercropping system did not imply the positive effect of interspecific competition between *B. striata* and Moso bamboo; instead, it was one of the competitive/invasive strategies of Moso bamboo (Chen et al., 2021). The Moso bamboo has considerable morphological and physiological plasticity in response to NH_4_^+^-N. Moso bamboo has significant growth and competitive advantage in NH_4_^+^-N-rich soil environments, which could further contribute to its growth rather than the crop species. These results could explain why the yield of Bletilla pseudobulb has a negative relationship with NH_4_^+^-N content. The shading effect may be another critical strategy of Moso bamboo to exclude intercropping species in interspecific competition (Lima et al., 2012). The rapid growth of Moso bamboo could increase the canopy closure of the stand in a short period, and our investigation showed that SRI in the PB intercropping system was the lowest among the three modes. Sunlight, a necessary factor for plant photosynthesis, significantly affects primary and secondary metabolism accumulation. The excessive lack of sunlight usually decreases the TCM yield (Guo et al., 2020).

There were no significant differences between all economic traits in CK and CB intercropping systems, which were related to mild interspecific competition between *C. paliurus* and *B. striata*. Although soil SOC, TN, and TP contents decreased, they did not differ significantly. Besides, the contents of soil NO_3_^-^-N and SWC were significantly increased. These results may relate to the growth habit of *C. paliurus*. As a deep-rooted deciduous broad-leaved tree species, *C. paliurus* could perfectly complement *B. striata* at spatio-temporal and functional under the intercropping. The difference in the spatial distribution of tree and crop roots was along with the soil profile. Tree roots distributed below the crop root zone could capture leached nutrients moving down the soil profile, typically for very mobile nutrients such as nitrate (NO_3_^-^), thus forming a “safety net” to reduce nutrient loss (Bergeron et al., 2011; Isaac and Borden, 2019). The safety-net process is associated with the “nutrient pumping effect,” which could acquire the mobile and weathered minerals in the deep soil profile and pump the nutrients to the litter tissue. The “new nutrients” were added to the surface soil *via* the decomposition of the litter. In addition, the SRI in the CB intercropping system was significantly lower than that in the monoculture, which does not mean *B. striata* in CB intercropping were under shading stress. The relationship between crop yield and light intensity was not linear, but an optimal light intensity range existed. Previous studies demonstrated that the *B. striata* were a shade-tolerant species, in which pseudobulb yield peaked at the canopy closure from 0.4 to 0.6 in the intercropping modes (Zhang et al., 2019). Notably, the advantages of intercropping were reflected in system unit productivity. Further research, such as the yield of *C. paliurus*, is needed to supplement to elucidate whether intercropping systems, compared to monoculture, have advantages in terms of overall system productivity.

### 4.2 Intercropping effects on the functional traits of Bletilla pseudobulb

Non-targeted metabolomics enables the unbiased detection of vast endogenous metabolites (Jorge et al., 2016; Ghatak et al., 2018). It can sensitively detect plants’ responses to the external environment compared with classical plant functional traits. Integrating the metabolites into functional ecology, under abiotic stress conditions, plants can activate signaling molecules such as jasmonic acid (JA), salicylic acid (SA), γ-aminobutyric acid (GABA), and abscisic acid (ABA), resulting in a series of protective mechanisms that lead to the accumulation and production of specific protective metabolites (Ghatak et al., 2018; Ali and Baek, 2020; Singh et al., 2020). These are diverse small molecular-weight osmoprotectants, including amino acids, amines, carbohydrates, polyols, and antioxidants (Obata and Fernie, 2012; Ghatak et al., 2018; Khan et al., 2020). Although the accumulation of these compounds was ubiquitous in plant cells, their concentration depends on the specific microenvironment, developmental state, and the types and duration of abiotic stress (Ghatak et al., 2018). In the present study, as we expected, the functional traits of Bletilla pseudobulb in the three modes were significantly separated, which differed from the findings of economic trait variations.

In CB intercropping, the content of raffinose, D-galactose, cellobiose, and D-ribose was significantly up-regulated. It has been documented that soluble sugars were involved in numerous biological processes and structural components of plant cells. In most plants, soluble sugars are the primary regulators of osmotic balance and accumulate significantly under stress (Sami et al., 2016), protecting plant cells as osmoprotectants (Sengupta et al., 2015). The main types of soluble sugars involved in plant stress responses were monosaccharides (glucose, fructose), disaccharides (sucrose, trehalose), raffinose family of oligosaccharides (RFOs), and fructans (Keunen et al., 2013). Interestingly, in the PB intercropping, the content of cellobiose, palatinose, melezitose, maltotriose, and D-ribose, instead of the common sugar osmoprotectants (D-fructose, D-glucose, trehalose, and raffinose), were significantly up-regulated. Zhang et al. (2018) reported that the response of plant sugar metabolism to different stresses was both convergent and divergent. In addition to RFOs and sucrose, the concentration of some rare sugars increased under stress. Although the concentrations of cellobiose, palatinose, melezitose, and maltotriose were previously observed to increase under abiotic stresses (Li et al., 2017; Xie et al., 2020; Li et al., 2022), the variation of these sugars among different species and under different abiotic stresses lacked commonality, especially under combined abiotic stresses. Carbohydrate metabolism plays a central role in regulating the functional traits of *B. striata*, and the mechanism of carbohydrate metabolism under intercropping deserves further research.

The amino acids, particularly branched-chain amino acids (BCAAs), played a central role in regulating the functional traits of *B. striata* in PB intercropping. Previous studies have reported that BCAAs such as valine, leucine, and isoleucine, as well as the amino acids that share synthetic pathways with BCAAs, including lysine, threonine, and methionine, could generally accumulate under abiotic stress (Joshi et al., 2010; Obata and Fernie, 2012). Amino acids contributing to abiotic stress may be related to scavenging reactive oxygen species (ROS), regulating intracellular pH, and osmoregulation (Rizhsky et al., 2004). The up-regulated BCAAs (L-valine, L-leucine, and L-isoleucine), methionine, L-lysine, and serine in PB intercropping mode may indicate that *B. striata* could better resist interspecific competition. Interestingly, there was no significant fold-change in proline content in this study, although it has been reported that proline content accumulates under the most stressful conditions (Kaur and Asthir, 2015; Meena et al., 2019). Joshi et al. (2010) reported a similar result, stating that plants accumulated more BCAAs than proline under abiotic stress. Regarding metabolic pathways, PB intercropping systems significantly affected the biosynthesis of amino acid metabolism in *B. striata*. Activating these metabolic pathways could improve plant tolerance to unfavorable external environments. For instance, Aminoacyl-tRNA biosynthesis was a critical pathway for the genetic information processing of translation. Aminoacyl-tRNA synthetases (aaRSs) could increase during stress and serve as a protein pool that directly participates in stress defense through noncanonical activities (Baranašić et al., 2021). Arginine is an essential medium for plant N storage and transport. Arginase (ARGAH) is the key enzyme in arginine metabolism. The expression of ARGAH is usually up-regulated under abiotic stress, which could improve plant N use efficiency and stress resistance (Shi et al., 2013).

Alterations in primary metabolism are the most common response of plants to abiotic stresses, including modifications in the levels of amino acids, sugars/glycols, and the tricarboxylic acid cycle. These metabolite variations exhibit common characteristics and vary by species and stress type (Singh et al., 2020). Our results indicated that *B. striata* under intercropping adopted different resistance strategies depending on the environmental stresses under different intercropping types. TN, SRI, and SOC were the dominant environmental factors in the change of functional traits of *B. striata* pseudobulbs under intercropping. Soil nutrient limitation dramatically affects plant growth and metabolism, especially soil macronutrients, such as C, N, P, and sulfur (S), which directly impact metabolism since most organic molecules comprise a combination of these elements (Obata and Fernie, 2012). The C starvation usually leads to an inhibition of plant growth. For example, organic acids and C-containing compounds, such as *Myo*-inositol, raffinose, glycerate, and fatty acids, were decreased in carbon-starved *Arabidopsis* seedlings (Osuna et al., 2007). In addition, photorespiration is inhibited when plants are exposed to prolonged shade, which also induces C-starvation. Jänkänpää et al. (2013) investigated the metabolic shifts in *Arabidopsis* seedlings grown under an illumination gradient. The soluble sugars (fructose, sucrose, glucose, galactose, and raffinose) were decreased under the low-light group, while most amino acids (arginine, asparagine, isoleucine, leucine, lysine, ornithine, tyrosine, and valine) affected by light conditions were accumulated under the low-light group. These findings are consistent with the metabolic shifts in functional traits of *B. striata* under PB intercropping. Interestingly, amino acid levels generally decreased under N deficiency due to the reduced synthetic substrates (Obata and Fernie, 2012; Hildebrandt, 2018). The increase in amino acid levels in the PB intercropping may be related to the decrease in protein synthesis and the increase in protein hydrolysis in *B. striata* under combinatorial stress. It is worth stating that the relative concentration of soluble sugars was not inhibited by light in the CB group while increasing as molecular osmoprotectants, mainly because *B. striata*, a shade-tolerant species, could tolerate moderate shade.

### 4.3 Correlation of economic traits with functional traits of Bletilla pseudobulb

Under most abiotic stress conditions, plant metabolism is usually disturbed, and the metabolic network must be reconfigured to maintain basic metabolism. Plants perceive stress through cell receptor-like kinase (RLK), which starts transcription factors and signal molecules to help plant hormones respond to stress conditions first (Yadav et al., 2016; Kosmacz et al., 2020). All these initial reactions involve synthesizing and degrading amino acids to produce primary metabolites such as carbohydrates, proteins, and lipids (Singh et al., 2020). The primary metabolism provides the necessary energy and precursor substances for the biosynthesis of secondary metabolites in the final step (Liu et al., 2017; Liu et al., 2021).

In this study, most of the functional node metabolites of PB were significantly correlated with economic traits and could reasonably predict the variation of economic traits. The sugar functional note metabolites (D-Glucose, Trehalose, and Maltose) in starch and sucrose metabolism were decreased and positively correlated with biomass. Starch is an essential factor mediating the abiotic stress response in plants, which typically consume starch to provide energy and carbon when plants are exposed to stress. The released sugars and other derived metabolites could act as regulatory osmolarity protectors to mitigate the effects of environmental stress (Thalmann and Santelia, 2017). Since starch is one of the main components of pseudobulbs of *B. striata*, inhibiting of the starch synthesis pathway under abiotic stresses of intercropping may further exacerbate the reduced yield of *B. striata*, which could explain the lower yield of *B. striata* in PB. The BCAAs and lysine showed a high fold increase under intercropping and were significantly positively correlated with total phenol and flavonoids, providing predictions for increased secondary metabolites in *B. striata*. It was documented that the BCAAs, aromatic amino acids, and lysine show exceptionally high fold increases under different abiotic stress (Obata and Fernie, 2012). However, this does not imply that BCAAs and lysine were directly involved in synthesizing secondary metabolites of *B. striata*. The aromatic amino acids (tyrosine, phenylalanine, and tryptophane) were considered precursors of a range of secondary metabolites (Tzin and Galili, 2010). As described in the previous section of the discussion, the increased relative levels of BCAAs acids and lysine may arise from the hydrolysis of proteins under the abiotic stress in intercropping (Hildebrandt, 2018).

All the functional node metabolites of CB were not significantly correlated with economic traits, which may be related to the low level of abiotic stress. Liu et al. (2020) comprehensively analyzed the primary-secondary metabolite network of wild and artificially grown ginseng. The results showed a stronger positive correlation between wild ginseng and secondary metabolite than artificially grown ginseng. It indicated that more primary metabolites were allocated to synthesizing secondary metabolites under complex environmental conditions in the wild plant. The cost of chemical defense in plants is considered to be expensive. Resources allocated to plant secondary metabolite synthesis, storage, and regulation will come at the cost of resources allocated to growth and reproduction (Stamp, 2003). Plants will preferentially produce secondary metabolites only if the defense benefit derived from their production is greater than the benefit derived from their growth (Byers, 2000; Barto and Cipollini, 2005). The differences in the correlation between CB and PB functional node metabolites and economic traits arise from the balance strategy between primary and secondary metabolism in *B. striata*.

## 5 Conclusions

In conclusion, the intercropping practice could alter the economic and functional traits of Bletilla pseudobulb. The PB intercropping significantly decreased the yield and significantly increased the total phenol and flavonoids of Bletilla pseudobulb, compared with CK. However, there were no significant differences in all economic traits between CB and CK. The functional traits among CB, PB, and CK were separated and exhibited significant differences. The *B. striata*, under intercropping, adopted different resistance strategies to the interspecific competition, in which *B. striata* adopted the strategy of up-regulating soluble sugars in the CB while adopting the strategy of up-regulating soluble sugars and amino acid compounds in the PB. The correlation between plant functional traits and economic traits depended on the environmental stress level. Plant functional traits in the PB group significantly affected economic traits and could accurately predict variation in economic traits. The environmental factors, Ns, SRI, and SOC, were the dominant controls in the variations of economic and functional traits. Therefore, proper management practices, including companion species density control and N fertilizer supplementation, should be taken to reduce the pressure of interspecies competition on *B. striata*.

## Data availability statement

The original contributions presented in the study are included in the article/Supplementary Material, further inquiries can be directed to the corresponding author/s.

## Author contributions

XX conceived the ideas and directed the project. PD and RY performed the field experiment and collected the relevant. XC, LC, HW, and PD performed experiment analysis. PD, RY, and HW wrote the manuscript. HW and LC improved it. All authors contributed to the article and approved the submitted version.

## Supplementary material

Supplementary Figure 1 The PLS-DA score plots and the permutation tests derived from GC-MS data. PLS-DA score plots **(A)** and **(C)** and the permutation tests **(B)** and **(D)** derived from GC-MS data of the Bletilla pseudobulb. The **(A)** and **(B)** indicates the *Cyclocarya paliurus* intercropping with *B. striata* (CB) *vs*. Control; **(C)** and **(D)** indicate the Moso bamboo intercropping with *B. striata* (PB) *vs*. Control.
