# Supplementary figures and images for: Comparative analyses of functional traits based on metabolome and economic traits variation of Bletilla striata: Contribution of intercropping
Source: Front Plant Sci. 2023 Mar 17;14:1147076. doi: 10.3389/fpls.2023.1147076 (PMC10064063; doi:10.3389/fpls.2023.1147076)

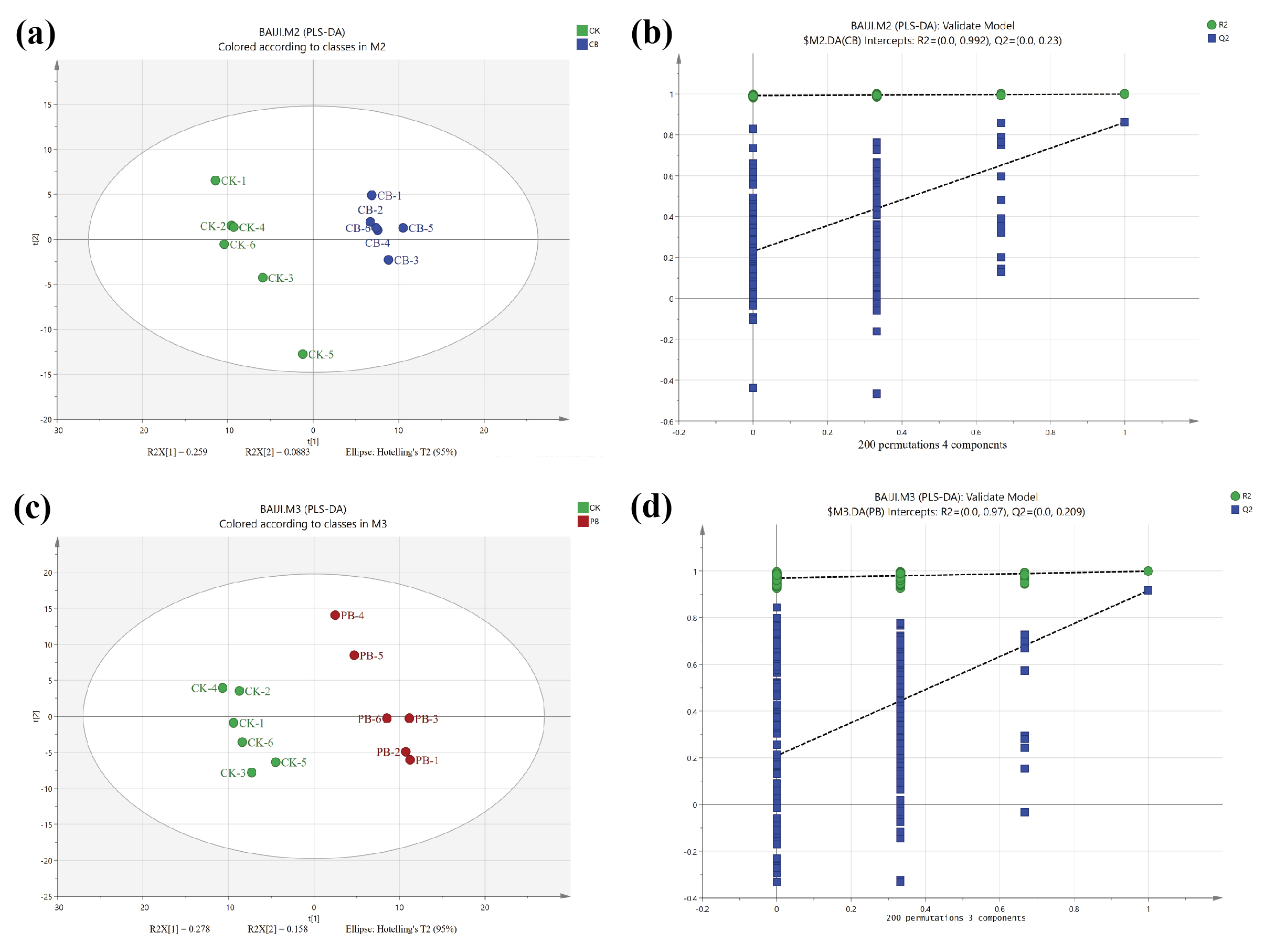

Supplement: Supplementary Figure 1 — The PLS-DA score plots and the permutation tests derived from GC-MS data. PLS-DA score plots (A) and (C) and the permutation tests (B) and (D) derived from GC-MS data of the Bletilla pseudobulb. The (A) and (B) indicates the Cyclocarya paliurus intercropping with B. striata (CB) vs. Control; (C) and (D) indicate the Moso bamboo intercropping with B. striata (PB) vs. Control. [file Image_1.jpeg]
